# Tutorial action during the veterinary doctoral program at the Complutense University of Madrid, Spain

**DOI:** 10.3389/fvets.2025.1564196

**Published:** 2025-04-02

**Authors:** Manuel Fuertes-Recuero, Juan A. De Pablo-Moreno, Ana Heras-Molina, Pablo Morón-Elorza, Teresa Encinas Cerezo

**Affiliations:** ^1^Complutense Veterinary Teaching Hospital, Complutense University of Madrid, Madrid, Spain; ^2^Department of Physiology of the Faculty of Veterinary Medicine, Complutense University of Madrid, Madrid, Spain; ^3^Department of Genetic, Physiology and Microbiology, Biology School, Complutense University of Madrid, Madrid, Spain; ^4^Department of Animal Production of the Faculty of Veterinary Medicine, Complutense University of Madrid, Madrid, Spain; ^5^Fundación Oceanografic de la Comunitat Valenciana, Valencia, Spain; ^6^Department of Pharmacology and Toxicology, Faculty of Veterinary Medicine, Complutense University of Madrid, Madrid, Spain

**Keywords:** tutor, thesis supervisor, veterinary students, PhD, doctorate

## Abstract

The tutorial activity within doctoral programs is a fundamental process for the comprehensive training of the doctoral student, encompassing more than just direct thesis supervision. In the context of the Spanish regulations (Royal Decree [RD] 99/2011) and the policies of the Complutense University of Madrid (UCM), the tutor's role is distinct from that of the thesis supervisor. The tutor is responsible for guiding the doctoral student in developing general, transversal and specific competences in the technical-scientific, teaching and academic fields, while the thesis supervisor focuses solely on overseeing and guiding the research work. This study examines the tutor's role in the Veterinary Doctoral Program, based on an interpretation of the guidelines established at the UCM and the perceptions of both tutors and doctoral students. It aims to analyze shared expectations and responsibilities in the tutoring relationship, to explore whether tutors meet students' expectations, and to understand tutors' approaches to their work. The methodology used is based on the analysis of documented agreements between students, tutors and thesis supervisors, as well as institutional regulations. The study emphasizes that tutorial action should not only include support in the field of research, but also a broader education. This holistic formation integrates complementary scientific training (participation in courses and conferences), aiding the student in teaching activities (preparation of classes, seminars or lectures), or helping in management activities such as applying for projects or grants. The results obtained highlight the fact that the tutor in the Veterinary Doctoral Program should evaluate and guide the students' training activities on an annual basis, fostering an open feedback relationship that promotes intellectual development and formative metaknowledge. This study emphasizes that tutoring should cover four areas: the scientific area, the teaching area, the personal area and the academic management area. In conclusion, the tutorial process should be a co-responsibility action between tutors and doctoral students who collaborate effectively, understanding and fulfilling their roles. Aligned with the doctoral program's goals, it is essential to support the academic and professional integration of the doctoral student.

## 1 Introduction

The doctorate is the highest level of academic training in most educational systems, designed to develop students' ability to generate new knowledge through advanced research. This academic program culminates in the awarding of a doctoral degree, which represents not only a profound mastery of a specific area of knowledge but also the capacity to contribute in an original and meaningful way to scientific advancement (1, 2). The doctorate extends beyond technical or theoretical training, encompassing the development of advanced competencies essential for assuming a prominent role in both the academic and professional communities (3).

Doctoral programs differ from bachelor or master degrees by emphasizing the generation of new knowledge through research rather than the learning of broad foundational education. These studies require the acquisition of advanced research skills to ensure scientific rigor and reliable results, as well as the integration of global and interdisciplinary perspectives, while aligning competences with career goals (4, 5). Doctoral students are expected to build a robust theoretical foundation, cultivate critical thinking, and develop the ability to contextualize findings within their social and scientific implications. Consequently, doctoral programs must equip students with a comprehensive understanding of their responsibilities and contributions as researchers (3, 6, 7). At the doctoral level, learning evolves into a reflective and introspective process where students not only internalize knowledge but also critically evaluate it and actively contribute to advancing their field of study (8, 9).

Transitioning from undergraduate to doctoral studies requires adapting to greater autonomy, understanding and navigating institutional policies, and developing strong self-management skills to meet the increased standards of responsibility (4). Furthermore, students often face emotional challenges, such as heightened expectations and feelings of isolation, highlighting the importance of effective time management, critical thinking, and the establishment of supportive networks, including mentors and peers (10).

For all these reasons, comprehensive doctoral training, which encompasses advanced competencies in research, teaching, critical thinking and leadership, requires institutional and academic support. The doctoral schools within each university, along with specific doctoral programs in the faculties often implement supervision systems for their doctoral training resources, involving multiple professors or researchers in various roles, such as mentors, directors or tutors. The primary objective of this system should be to provide tools that facilitate doctoral students' adaptation and foster positive experiences through the doctoral itinerary (7).

This policy review examines the role of tutors in the Doctoral Program in Veterinary Medicine at the Complutense University of Madrid (UCM). It analyzes the current regulations governing the program and conducts a comprehensive comparison with other doctoral programs. The review considers perspectives from both doctoral students and thesis tutors and directors to ensure well-rounded support that effectively addresses students' academic and professional training needs within the current academic and scientific landscape. Based on this analysis, recommendations will be proposed for the direction of tutorial practices, aiming to provide comprehensive support that effectively addresses students' training and professional needs within the current academic and scientific context.

## 2 Comprehensive doctoral training in the veterinary program of the UCM

The main aim of the Veterinary Doctoral Program of the UCM, is to provide its students with different abilities to design and develop research studies in the fields related to veterinary sciences, both basic and applied such as biology, animal physiology, biochemistry, animal production and reproduction, animal pathology, pharmacology, toxicology, among others. To achieve this objective, there are adhere to the Doctoral Program 29 research groups, which also collaborate with different institutions worldwide (11). This Doctoral Program was approved in the Resolution of February 28, 2014, of the General Secretariat of Universities, publishing the Agreement of the Council of Ministers of February 21, 2014, which establishes the official status of certain Doctorate degrees and their registration in the Registry of Universities, Centers, and Degrees (12). As other Doctoral Studies of the UCM, the Veterinary Doctoral Program is regulated by the RD 99/2011.

Although the final milestone of veterinary doctoral studies is the presentation and defense of the doctoral thesis, the integral formation and development of the doctoral student extends far beyond the completion of this work. This training should encompass comprehensive development in research, teaching, socialization, and university management, essential components for building a successful academic or research career. Effective integration into the academic community relies not only on conducting rigorous research but also on fostering collaboration, communication, and adaptability to institutional structures.

Outlined below are the key competences that doctoral students need to acquire, identified through research involving the perspectives of students, teachers and educational administrators involved in the veterinary doctoral program. These competences reflect the multifaceted demands of academic and scientific careers and highlight the importance of a balanced, integrative approach to doctoral training

Scientific competencies include those that are strictly technical, such as learning to design experimental protocols, project development, and mastering clinical or laboratory methods and techniques. They also include other essential aptitudes, such as the ability to critically analyze the literature and available information. These skills enable students to rigorously evaluate both their findings and the literature. This process requires advanced competencies of evaluation, interpretation and reflection that extend beyond theoretical and methodological knowledge. In this area, it is also important that the training is aimed at providing the student with the ability to develop scientific activity autonomously.

Furthermore, doctoral training in veterinary research should enable students to assume leadership roles within their field of specialization, allowing them to actively contribute to knowledge creation through innovation. In addition, prioritization and enhancement of leadership skills, enable students to effectively manage projects and lead teams within their areas of expertise This approach also fosters an awareness of the ethical and social implications of their contributions, enriching both their research endeavors and their professional identity (6, 9, 13). Equally important is the promotion of intellectual growth, which stems from the continuous application of critical analysis and evolves into the development of creativity and innovative capacity. Finally, these efforts should culminate in a heightened sense of ethical and social awareness, ensuring that students understand and address the implications of their contributions to both science and society (6, 13).

Formal structures like the veterinary doctoral program provide essential resources and environments for skill development, while informal networks, including peers and mentors, foster collaboration and emotional support. As mentors, professors and researchers contribute by integrating both theoretical and practical knowledge, connecting students to scientific networks, and aiding their academic and professional integration. Support from fellow doctoral students helps mitigate feelings of isolation, fostering shared experiences and a sense of belonging that is crucial for persistence and success (4, 14). All these abilities related to the development of critical thinking, social growth and leadership that are cultivated in the scientific domain have far-reaching applications beyond academia. These competencies represent personal learning experiences that enable individuals to distinguish themselves in different scenarios, from problem-solving in organizational settings to decision-making in complex and dynamic environments. By fostering adaptability and innovative thinking, these skills ensure that doctoral students are prepared to make meaningful contributions across a wide range of professional and societal contexts.

## 3 The figure of the tutor according to the regulations in the doctoral program at the Complutense University of Madrid, Spain

In Spain, RD 99/2011, which regulates the Veterinary Doctoral Program of the UCM, establishes specific recommendations regarding the competencies doctoral students should acquire, highlighting the importance of supervision and tutoring as fundamental elements in their academic development. Previous studies have identified insufficient support and follow-up systems as key factors contributing to doctoral attrition, as feelings of disorientation or isolation can diminish motivation and increase the likelihood of students dropping out (8). Consequently, effective supervision and comprehensive support structures are essential for ensuring student persistence and success throughout doctoral training (15–17).

In some Spanish universities, three key roles are involved in doctoral supervision: the thesis supervisor, the academic tutor, and the mentor. Each of these figures plays a distinct but complementary role in supporting the doctoral student throughout their training. The thesis supervisor provides direct guidance on research, supporting the development and execution of the doctoral project. The academic tutor monitors the student's overall progress, ensuring compliance with educational and administrative requirements. The mentor offers professional and personal support, facilitating the student's integration into the scientific community. While the concept of a doctoral mentor is less common compared to a scientific mentor, it is also found in other countries, such as Taiwan, where mentors play a key role in supporting doctoral students both academically and professionally (18). In many other countries, the specific role of a doctoral tutor, as defined in the Spanish system, is not explicitly recognized. However, similar responsibilities are often incorporated into the role of the thesis supervisor or shared among advisors, co-supervisors, or members of a doctoral committee (17, 19). Regardless of how these roles are organized or assigned, it is crucial that they are clearly defined. A lack of clear differentiation between these roles can result in inconsistent supervision, negatively impacting the doctoral student's experience (8, 16).

**Figure 1:**
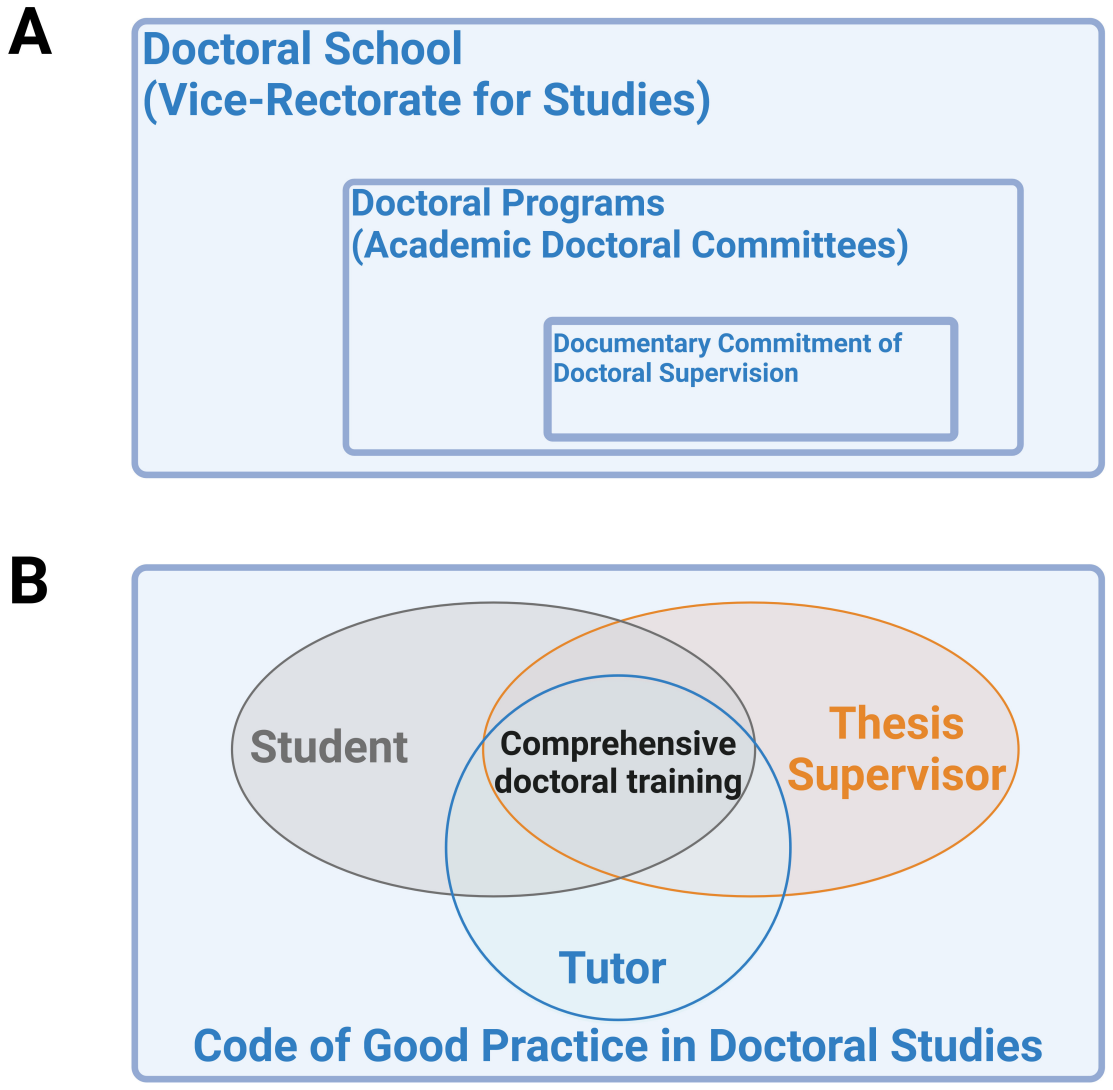
Complutense University of Madrid (UCM) doctoral teaching systems. **(A)** Formal academic structures. **(B)** Figures involved in practice.

Within the Spanish regulatory framework, in the UCM, the general supervision of doctoral training is managed by the Academic Committee, an entity appointed for each Doctoral Program, including Veterinary Doctoral Studies (Figure 1A). This Committee annually monitors compliance of both the doctoral candidate and their supervisors with the training and experimental activities. The Academic Committee recognizes two key figures in the doctoral supervision process, ensuring that their roles and responsibilities are effectively fulfilled: the thesis supervisor and the tutor. The tutor, as outlined in the RD 99/2011, fulfills the combined functions of the academic tutor and mentor, as described in the previous paragraph. According to the regulations, the tutor must be a university teacher with experience in the academic field. While the tutor may also serve as the thesis supervisor, the roles do not necessarily have to be filled by the same person, allowing for greater flexibility and diversity in the support the doctoral student receives (Figure 1B). In cases where a single person assumes both roles, dynamic supervision can become complicated, potentially affecting the quality of guidance and support provided to the student (16).

The Code of Good Practice in Doctoral Studies of the Complutense University of Madrid (UCM-CGPDS) further elaborates and clarifies the role of the tutor in the context of doctoral supervision (20). This document establishes that the tutor and the thesis director(s) together form the supervisory team, whose collective responsibility is to guide the doctoral student in the academic, ethical and administrative aspects of their research. The team must work in close coordination under the leadership of the thesis director, who is accountable for overseeing the group's efforts and ensuring that the contributions of both the tutor and thesis directors are consistent with the overall objectives of the doctoral project. According to the UCM-CGPDS, this coordinated approach aims not only to guarantee the quality of the thesis, but also to provide the doctoral candidate with a comprehensive training environment that facilitates their integration into the academic and scientific community (Figure 1B). Moreover, this approach should enable students to design and execute complex research projects, using appropriate tools and integrate ethical and critical practices to produce impactful and meaningful research (16).

The profile of an ideal tutor, according to the UCM-CGPDS, focuses on academic guidance and the ethical and professional support of the doctoral student. The tutor must have substantial research experience, preferably in a different department from that of the thesis supervisor, ensuring a broader perspective in the student's training. The tutors play a key role in ensuring that the student's thesis development aligns with the objectives of the doctoral program. Their responsibilities include monitoring the student's progress, providing adequate resources, producing annual reports, and mediating the relationship with the thesis supervisor to avoid any potential conflict. In addition, the tutor is expected to promote ethical values, encourages the student's active participation in the academic community, and prioritize the holistic development of the student throughout the doctoral process (20).

A recent study highlighted a strong interest among doctoral supervisors in receiving formal training in supervision, recognizing the need to enhance their skills. Some supervisors have indicated that they did not know exactly what they needed, but acknowledged the value of gaining a fundamental understanding of their responsibilities (21). In some academic environments, networks and groups of tutors have been created to organize regular meetings where supervisors can share their experiences. These interactions contribute to the continuous improvement of supervision practices and enrich the overall doctoral supervision process (6, 22).

The Documentary Commitment of Doctoral Supervision (DCDS), which is included in the RD 99/2011, is the official document that comprises the supervision process within the doctoral program (Figure 1A). It must be signed by the Academic Committee responsible for each UCM doctoral program (such as the Veterinary Program), the doctoral student, the thesis supervisor and the tutor, and remains valid from the date of signature until the thesis is defended (23). In this document, the doctoral student commits to carrying out the doctoral studies and research that are the subject of the thesis project within the framework established by the applicable regulations. This commitment is made under the supervision of the tutor and thesis supervisor, and the student agrees to follow the guidance provided by both regarding the completion and monitoring of training and research activities. Additionally, the student commits to engaging in spontaneous learning experiences that help develop research, management and critical thinking skills organically, facilitating their integration into the academic community and promoting their autonomous and professional development (20, 24, 25).

The tutor agrees to adapt the training and research activities of the doctoral candidate to align with the principles of the program, as well as to approve, supervise, and regularly monitor the personal training plan and related activities. The tutor is also responsible for submitting annual evaluation/monitoring reports to the Academic Doctoral Committee. Additionally, the tutor provides the necessary guidance and advice, ensuring that the student develops initiative and successfully integrates into the doctoral program. The thesis supervisor(s), up to three of whom may be designated, commit to supervising and regularly monitoring the personal training plan and the research activities developed by the doctoral candidate. They are responsible for providing the necessary guidance and advice to ensure that the candidate develops initiative and achieves autonomy in the research task. Furthermore, the thesis supervisor(s) commit(s) to supervising and reviewing the doctoral thesis until its completion.

If the doctoral candidate is unable to find a tutor and/or supervisor, the Academic Committee is responsible for assigning a tutor and/or supervisor to each doctoral candidate admitted to the program. In the event of non-compliance, the Committee will consider the possibility of changing the tutor and/or supervisor. Furthermore, if a conflict arises due to non-compliance with any of the terms outlined in this commitment, the parties involved agree to submit the matter to the decision of the Academic Committee of the Doctoral Program as the first course of action (23).

## 4 The tutor-doctorate relationship

The relationship between the tutor and the doctoral student is a central component in the veterinary doctoral training process, characterized by an interpersonal interaction based on mutual trust and orientation toward clearly defined objectives (26). According to the UCM-CGPDS (20), this relationship not only enables the academic development of the doctoral student but also has a significant influence on their integration into the scientific community and on their motivation and persistence in the program. The matching of personalities and expectations between tutor and student is crucial for the success of this relationship (27). From the outset, the establishment of a strong relationship can be facilitated by a pre-enrolment interview, where both the student's academic goals and the tutor's expectations and supervisory style are explored (28). Throughout the doctoral process, frequent meetings help reinforce this dynamic, providing continuous opportunities for evaluation and adjustment of research objectives. In addition, although the relationship is primarily academic, cultivating a moderate personal bond can strengthen trust and provide opportunities for empathy and understanding during times of difficulty for both the student and tutor (20, 29).

Each member gives essential elements to this relationship, creating a dynamic and reciprocal interaction. The doctoral student actively contributes by asking for advice, guidance and support in specific areas of their research project. They also communicate their needs regarding methodological skills and expertise, which helps share the tutor's approach. Through continuous feedback, the student allows the tutor to adjust their guidance and support according to the student's progress and difficulties. Tutors, in turn, provide critical methodological suggestions and strategic research guidance, sharing their expertise and accumulated knowledge in the field. They also facilitate the students' integration into academic networks, give them a broad vision of the disciplinary context and encourage the publication and dissemination of their results (20, 23). The relationship between tutor and doctoral student becomes, therefore, a fundamental axis of the doctoral process, the success of which depends on open communication and reciprocity, which enhances both the academic and personal development of the student.

Regarding the relationship between tutor and doctoral student in the veterinary doctoral program, it is important to find a balance that avoids excessive dependency and encourages the student's independence. The development of autonomy is a central objective of doctoral training, and the tutor has a key role in providing guidance that enables the doctoral student to progress toward self-sufficiency. Haley et al. highlight that it is an ethical aspect of supervision to provide room for doctoral students to make their own decisions in research, facilitating their development as future independent researchers (30). Additionally, Zackariasson and Magnusson analyze how supervision can be designed to contribute to the student's learning and development, emphasizing the importance of practices that promote academic independence (31). According to the UCM-CGPDS, effective guidance should empower the doctoral student to evolve into a confident researcher capable of making informed decisions and managing their research project. Supervision must avoid over-involvement that could limit the student's autonomy and critical thinking capacity. Instead, it should provide the support and tools necessary for the student to navigate challenges and grow both academically and professionally (20).

One of the most common problems in the tutor-doctoral student relationship is the physical unavailability of the tutor, often due to external professional commitments or academic stays. This lack of availability can leave the doctoral students feeling isolated or disorientated, potentially affecting the continuity of their research and diminishing their motivation. Another common difficulty is a lack of personal understanding between the tutor and the student, which may result from differences in working or communication styles and approaches. Such conflicts, if not properly addressed, can affect the effectiveness of supervision and hinder the progress of the doctoral thesis (20, 21).

To identify these problems and improve the working dynamic, regular evaluations of the relationship between the tutor and doctoral student are essential. These evaluations provide an opportunity to analyze both successful aspects and areas requiring improvement in the interaction, as well as the progression of the thesis. Such process fosters mutual learning and ensures a constant adaptation of the supervision to the needs of the doctoral candidate. The inclusion of meta-knowledge analysis, i.e., the understanding that the doctoral students and the tutor have of their own learning and supervision processes, is a valuable resource for identifying areas requiring improvement in communication and expectation management. Finally, these evaluations should also lead to suggestions for improvement to ensure a supervisory environment that maximizes the doctoral student's potential and promotes their growth and development toward full independence across all aspects of their academic training (20, 26, 32).

## 5 The tutorial action

The tutorial action in the context of doctoral studies is conceived as a set of strategic and collaborative efforts proposed by the doctoral student and the tutor to fulfill the essential functions of tutoring. This process of two-way interaction has been described as a “knowledge relationship” in which both actors (tutor and student), actively contribute to the co-construction of knowledge and the development of key competencies that enable and empower the student to progress comprehensively in their academic and professional training (16, 20).

The doctoral tutor's actions should be integrated into the daily academic and scientific life, taking advantage of real opportunities such as seminars, meetings, and research activities that surround the doctoral student's activity. This approach, described in the UCM-CGPDS, promotes flexible and contextualized supervision in which the tutor guides the doctoral student in an immersive learning process without imposing artificial constraints. Collaboration between universities, similar to the framework implemented in WNGER II in Norway, can address shared challenges in doctoral education, optimize resources, and enhance the quality of supervision (33). Applying similar collaborative and interdisciplinary strategies to the analysis of tutorial activities at UCM could strengthen the argument that a collaborative and interdisciplinary approach is essential to enhance the holistic education of doctoral students.

The tutorial action should take place at four levels, further discussed in this section: scientific, teaching, academic management and personal (15, 34). At the scientific level, the tutor guides the doctoral student in the methodology and rigor of research, ensuring that the student acquires and applies in-depth knowledge in his/her field. At the teaching level, the tutor develops the doctoral student's pedagogical skills and prepares him/her for academic and teaching responsibilities. At the academic management level, the tutor guides the students in dealing with administrative tasks, organizing their work, and participating in university life. Finally, at the personal level, the tutor provides both emotional and professional support, helping the doctoral student to develop resilience and interpersonal skills to navigate the challenges of the doctoral journey effectively (4, 25). Table 1 shows a summary of these levels as described below in this article.

**Table 1:** Summary of the tutor's activities categorized into four levels of tutorial action.

**Tutorial action**			
**Scientific level**	**Teaching level**	**Personal level**	**Academic management level**
Integrate into a research group. Introduce the student to other scientists. Invite and guide in attendance at scientific events. Stimulate the search for information and scientific updating. Encourage learning new methodology. Promote training stays in other institutions. Motivate and promote perseverance and scientific curiosity.	Integrate into teaching groups. Stimulate participation in teaching innovation projects. Invite to actively participate in teaching activities (lectures, practices, seminars). Guide in the planning and preparation of teaching material. Encourage attendance and participation in educational events (courses, congresses, conferences, etc.).	Mutual and dynamic emotional support. Provide tools to manage stress related to thesis work. Listen, share and seek solutions to personal or study-related concerns. Encourage relationships with peers and participate in networks of doctoral students. Support economic stability through guidance and assistance in applying for financing.	Guide in the completion of the doctoral administrative forms. Promote updating in scientific and educational policy. Advise and guide in the application for scholarships and research projects. Direct and advise on the development and configuration of the curriculum vitae. Advise on the management of public events.
Promote autonomy in conducting activities related to the doctorate (research, teaching, management, etc.) Encourage entrepreneurial and leadership skills by providing opportunities to organize various activities.			

The extension of tutorial action to the last three levels marks a conceptual difference between the terms “doctorate” and “PhD” (Philosophiae Doctor). While a doctorate, in its more traditional definition, may focus exclusively on scientific research to complete a thesis, the “PhD” integrates a broader training that includes interdisciplinary and managerial skills and prepares the researcher for a comprehensive performance in the academic and professional field. In this sense, both terms can be used interchangeably when the tutorial action is comprehensive and covers all levels of performance, reflecting the contemporary view of doctoral training as a global process that forms not only specialists, but also critical thinkers and research leaders, as occurs in the UCM (6, 8, 24).

Other authors have described the Integrated Competing Values Framework (ICVF) model as a tool to explain how the supervisors (thesis supervisor and/or tutor) can adopt different roles (Developer, Deliverer, Monitor, Broker, Innovator) organized around a central integrative role that ensures balance among these functions in supervisory process. This approach can support the argument that mentoring should be flexible and adaptable to the evolving needs of the learner (35).

Regardless of the specific model or framework for tutorial action, research consistently highlights a clear link between the quality of the supervision provided by the tutor and the success in the doctoral career (36).

### 5.1 Scientific level

Within the framework of doctoral training, it is assumed that doctoral programs, including veterinary programs, comply with the established regulations, particularly those ensuring the provision of adequate resources for conducting research. These include financial, material, and technological support, which are essential for the doctoral student to execute and develop their project effectively. Ensuring these resources prevents disruptions that could hinder the student's academic and scientific progress (20, 25).

In addition to providing resources, the tutor must promote the professional development of the doctoral student on several levels. One of the key areas is the facilitation of personal contacts and integration into related research groups. This includes inviting the doctoral student to participate in scientific events, such as seminars and laboratory meetings, which help them build networks within the academic community. Participation in scientific networks not only strengthens their research profile but also cultivates transversal skills, including project management and an understanding of institutional dynamics (4).

Similarly, the tutor has the responsibility to inform and guide the student toward scientific events of formative interest, such as courses, congresses, and meetings. These events not only broaden the doctoral student's knowledge and skills, but also expand their academic and professional network (8, 16, 37, 38). To cultivate specific scientific competencies, the tutor should direct the doctoral students toward training opportunities that develop skills in areas such as searching, reading, and writing scientific texts; developing scientific reasoning; mastering new methodologies and advances in their field; and data processing. This guidance should be accompanied by a focus on autonomy, encouraging the doctoral student to independently identify and utilize these resources and tools, which is crucial for his/her development as an independent researcher (4, 6).

It is essential that the tutor, in guiding the student, does not interfere with the functions and responsibilities of the thesis supervisor, especially when these two figures are not in the same person. This means that the tutor's role should be limited to offering guidance and support without directly intervening in the researcher's development and technical evaluation. This approach promotes balanced supervision, providing the doctoral student with comprehensive support while safeguarding their autonomy and respecting the role of the director (20).

Finally, motivation and encouragement for research are fundamental aspects of the tutor's action. The tutor must cultivate in the doctoral student a positive and proactive attitude toward the research process, promoting an environment that values intellectual curiosity and perseverance. This emotional and motivational support not only contributes to the success of the research but also strengthens the doctoral student's commitment to their academic and scientific vocation (6, 22). Furthermore, effective supervision fosters independence in research while simultaneously developing essential skills such as leadership, self-management, conflict resolution, and problem-solving skills, critical for integration into scientific communities and environments (16, 21, 39).

### 5.2 Teaching level

The teaching dimension of the veterinary doctoral tutorial action, although not essential, can be developed when the doctoral student's interests are oriented toward an academic career rather than a career in the business or technology sector. This aspect becomes an important formative opportunity for doctoral students who wish to integrate into the university environment, allowing them to develop basic teaching skills and enrich their professional experience within an academic context (4, 20). A committed tutor can encourage the doctoral student's participation in teaching activities within the department or research setting where their thesis is being conducted, if these activities align with the student's academic interests. This participation, which may include preparation for and participation in classes, seminars, and practical sessions, will allow the doctoral student to become familiar with the pedagogical aspects and teaching dynamics within their discipline (8, 37, 38).

In addition, the tutor has an important role in guiding teacher training opportunities. This includes informing the doctoral student about courses in didactics, educational innovation projects, and teaching conferences, which may or may not be related to the tutor's academic activity. Through these opportunities, the doctoral student can acquire essential skills for university teaching, such as searching, reading, and writing pedagogical texts, developing critical thinking, and implementing new teaching methodologies that respond to current challenges in higher education (6, 24, 25). It is equally important to encourage the doctoral students' autonomy in this area, directing them toward the necessary resources to independently identify the spaces and activities that will help them enhance their teaching skills. This guidance helps the doctoral students to acquire a critical and constructive approach to their teaching process, enabling them to design, implement, and engage in effective learning sessions. These experiences not only strengthen the doctoral student's academic skills but also prepare him or her for public speaking engagements, report writing, and future roles of greater teaching responsibilities (16, 20). Therefore, the teaching dimension of the tutorial process serves as a complementary development path that, by enhancing the teaching and pedagogical skills of doctoral students, broadens their professional profile and strengthens their preparation for an integral academic career.

### 5.3 Personal level

The personal dimension of the tutorial action within the doctoral program addresses emotional, economic, and social aspects to promote a comprehensive formative experience for the doctoral student. At the emotional level, the personal and academic relationship between the doctoral student and the tutor is considered fundamental, since an interaction based on trust and empathy can strengthen the students' resilience and adaptability to the demands of their training. According to the UCM-CGPGS (20), the tutor can play an active role in providing tools to help the doctoral student cope with the stress of the academic workload and family pressures, in addition to enhancing self-motivation and leadership skills. This two-way relationship promotes open communication, allowing the doctoral student to express concerns and difficulties. This mutually supportive dynamic strengthens emotional stability and enhances the effectiveness of the student's research development (8, 20, 21, 40, 41).

The economic situation is a crucial factor in the doctoral experience, as financial security allows the doctoral student to focus fully on his/her research (42). In this context, the tutor can act as a facilitator, guiding the doctoral student through the various scholarship opportunities and personal funding program available, both nationally and internationally. This guidance not only increases the chances of obtaining financial resources but also helps the doctoral student develop essential skills for managing their academic career, strengthening their capacity for planning and foresight (25, 40). In addition, support from fellow doctoral students helps mitigate isolation, promoting shared experiences, creating a sense of community. This sense of belonging crucial for maintaining motivation and achieving long-term success (4, 14).

From a social point of view, relationships with other doctoral students are vital for both academic and personal development. Participation in working groups and academic networks, including interdisciplinary contexts, favors the exchange of ideas and the creation of a collaborative learning environment. The tutor can promote this social integration by facilitating participation in seminars, discussion groups, and interdepartmental meetings, thus strengthening their connections within the academic community.

Therefore, the tutorial action in the personal sphere not only supports the doctoral student's development as a researcher but also addresses their emotional wellbeing, economic stability, and social integration. This holistic approach strengthens doctoral training by equipping the doctoral students to face the challenges of their training with a balanced and resilient vision.

### 5.4 Academic management level

Although the tutorial action on academic management extends throughout the entire doctoral period, it is particularly relevant at the beginning of the training and at the point of completion, when students submit their doctoral thesis. During the initial stages, guidance in processes such as pre-enrollment, enrollment, and supervisor assignment is essential to ensure that the doctoral student is properly integrated into both the doctoral program and the institutional structure. Similarly, collaboration in planning training activities and conducting the annual review of the research plan are essential tasks at this level. In this process, the tutor plays a guiding role by proposing activities and training requirements, while the doctoral student carries them out, and the tutor ultimately evaluates them. This ensures a structured and effective approach to monitoring the student's progress (16, 20, 43, 44).

In addition to the functions directly linked to the doctoral program, the tutor also plays a key role in supporting other administrative aspects, such as the doctoral students' participation in institutional forums (Departmental Councils, Faculty Board, and Student Associations), which helps their active integration in the Faculty of Veterinary Sciences and the UCM. Likewise, the tutor guides the doctoral student in understanding the university's teaching and research organization, keeping them informed about new developments in university policy or research. This guidance facilitates their adaptation to any regulatory or structural changes that could influence their academic development (8, 20, 25).

Support in academic management also extends to the extra-university sphere, where the tutor guides the doctoral student in areas such as applying for financial support (grants and pre-doctoral contracts) and identifying research projects both within and outside the university environment. This support also includes providing curricular guidance to help the doctoral student integrate into academic and professional networks. The tutor also advises on registering and submitting papers to conferences, scientific societies and academic journals, which is key for the student's projection in the scientific community and recognition in their research field (4, 6, 44). In addition, the tutor can guide the doctoral student in the organization of scientific events such as seminars, conferences, and meetings of scientific societies, thus providing valuable experience in planning and coordinating academic activities. These management skills not only enhance the doctoral student's professional profile, but also foster leadership and collaboration skills in collective projects, which are highly valued aspects in academic and scientific careers (20, 22).

Academic management is, therefore, an integral dimension of the doctoral tutorial action, providing the doctoral student with the tools and support needed to navigate effectively in the institutional, academic, and extra-academic environments. This approach promotes a complete and comprehensive doctoral experience in which the doctoral student also develops administrative and management skills that are essential for their future career (42).

## 6 Conclusion

In conclusion, the tutor plays a crucial role in the comprehensive training of doctoral students, addressing various dimensions essential for their academic and professional development. The UCM regulations consider that effective tutorial action should be rooted in a relationship of trust and co-responsibility, enabling open communication and continuous feedback to adapt to the student's progress and evolving needs. Beyond guiding the dissertation project, the tutor must nurture skills in research, teaching, management, and personal growth, preparing the doctoral student to become an autonomous, critical, and socially committed professional. Therefore, the tutor, as a key element in the holistic approach to comprehensive doctoral training, should not only ensure the successful completion of the thesis but also equip the student to face the challenges of an academic and scientific career. Based on our experience, many of the activities outlined in the four level of tutorial action are seldom implemented by the majority of tutors at our institution. To improve tutorial action, it would be necessary to provide the university faculty with better information about the tutor's functions and responsibilities. Additionally, providing courses to the tutors that cover the four aspects of tutorial action could significantly improve this activity.
